# Internet addiction and mental disorders: Clinical effects by self-distancing

**DOI:** 10.1192/j.eurpsy.2021.95

**Published:** 2021-08-13

**Authors:** G. Bersani

**Affiliations:** Department Of Medico-surgical Sciences And Biotechnologies, Sapienza University of Rome, Latina, Italy

**Keywords:** Internet addiction, covid 19, psychopathology

## Abstract

We face today the huge and very rapid worldwide growth of behavioural issues related to the use of Internet. The definition of Problematic Internet Use (PIU) refers to new behavioural patterns that can potentially affect in variable degree, from mild to extremely severe, both individual and social wellbeing. PIU is strongly increasing in people affected by different forms of mental disorders and personality disorders, often inducing substantial changes in their clinical phenomenology, with consequent emergence of new symptom and course profiles. On the other side, PIU represents itself with growing frequency as a factor with high potential of inducing progressive psychological and behavioural impairment, with possible negative outcome on personal and psychosocial wellbeing and adjustment, also potentially leading to the development of new specific forms of psychopathology. Among PIU patterns, Internet Addiction (IA) plays a central role, due to its wide diffusion and behavioural as well as interpersonal and social consequences. The worldwide COVID 19 epidemics induced limitations in direct social relationships, such as social distancing, appear to lead to changes of patterns of IA, for an increase of time spent in addictive behaviour and a further reduction of research of interpersonal contacts. Obsessive-compulsive and autistic-like behaviour are differently reinforced by the combined effect of compulsory self distancing and general health concern, but also possibly induced in previously not affected subjects. Anxiety and mood reactivity also contributes to maladjustment profiles. Further evidences and new guide-lines are requested to face this novel and multifactorial social and clinical phenomenon.

**Disclosure:**

No significant relationships.

